# Supplementary material for: Are carbon emissions trading and green financial instruments synergistic? -Comprehensive quantitative research based on content analysis
Source: PLoS One. 2024 Mar 7;19(3):e0298601. doi: 10.1371/journal.pone.0298601 (PMC10919601; doi:10.1371/journal.pone.0298601)
Supplement: S1 Data — (ZIP) [file pone.0298601.s001.zip › Data/地级市Z_F工作报告.zip!/╦─┤¿/├α╤⌠╩╨/└┤╫╘╣1⁄2╓┌║┼í╛┬φ┐╦╩2╛▌═°í┐_2017.docx]

各位代表：

现在，我代表市人民政府向大会报告工作，请予审查，并请市政协委员提出意见。

过去五年工作回顾

过去五年，是绵阳发展历程中不平凡的五年，也是经济社会发展取得新成就的五年。五年来，在党中央国务院、省委省政府和市委坚强领导下，在市人大、市政协监督支持下，我们紧紧依靠全市人民，主动适应经济发展新常态，积极应对各种困难挑战，完成了本届政府既定的各项目标任务，开创了经济社会发展的新局面。

——这五年，我们坚持科学发展、加快发展，综合实力迈上新台阶综合实力明显提升。地区生产总值年均增长11.2%、达到1700.3亿元，全社会固定资产投资年均增长7%、达到1154.1亿元，地方一般公共预算收入年均增长18.2%、达到104.1亿元，城乡居民人均可支配收入年均分别增长12.2%、13.7%，达到27170元、12349元。先后荣获或蝉联全国文明城市、全国科技进步先进市、全国创业先进城市、国家知识产权示范城市、全国人民防空先进城市、国家卫生城市、国家森林城市等殊荣，城市综合实力、影响力和美誉度不断提升。

三次产业协调发展。产业结构由17.3:48.8:33.9优化为15.3:50.5:34.2。规模以上工业增加值年均增长12.8%。高新技术产业和战略性新兴产业产值占工业总产值比重分别提升到52%、34%。服务业增加值年均增长9.7%。社会消费品零售总额年均增长15.2%、达到879.2亿元。电子商务交易额稳居全省第2位。旅游总收入年均增长37.4%。农林牧渔业总产值年均增长4%。建成现代农业万亩示范园76个，高标准农田突破200万亩。

城乡建设成效显著。“5·12”汶川特大地震灾后重建任务胜利完成。安县成功撤县设区。中心城区建成区面积增加36.1平方公里、达到139.1平方公里，常住人口增加25.8万人、达到132.8万人。城镇化率提高8.15个百分点、达到48%。畅通绵阳建设取得积极进展，二环路一二期、机场直通道等市政工程建成投用。成绵乐城际铁路、绵遂高速、成绵高速复线、成德南高速、绕城高速南环线建成通车。全市公路通车里程突破2万公里。绵阳机场通航城市达到26个，旅客年吞吐量突破150万人次。完成水利建设投资148.8亿元，武都水库、燕儿河水库建成投用。实施农村饮水安全工程，受益群众达到175.3万人。能源信息设施水平不断提升。建成国省重点镇示范镇21个，幸福美丽新村585个。

——这五年，我们坚持创新驱动、军民融合，科技城建设开创新局面

自主创新能力显著增强。建成国家、省级企业技术中心66家，国家、省级工程技术研究中心19个，国家、省级重点实验室25个。“千英百团”聚才计划*实施，中国国际人才市场国家科技城（绵阳）分市场成立运行。申请专利23985件，获得专利授权13355件。研究与试验发展经费支出占地区生产总值比重达到7%，高新技术产业化指数达到79.6%，科技进步综合水平指数达到68.28%。空气动力新城、航空新城基本建成，科学新城建设加快推进。

科技成果转化持续加快。创新中心二期、软件产业园等创新转化平台功能逐步完善。工业技术研究院、中科创新育成中心、技术转移中心建成运行。全市科技孵化器达到50个，总面积达到90万平方米。中国创新驿站绵阳工作站、中国技术交易所绵阳工作站作用有效发挥。实现技术合同成交总额28.9亿元、年均增长10%。组建新能源汽车、北斗卫星导航等产业技术创新联盟15家。“涌泉计划”*推进，累计认定科技型中小企业8470家。

军民融合发展*推进。被确定为国家军民融合科技创新示范基地，纳入国家系统推进全面创新改革试验区域。全国唯一的国家军民两用技术交易中心挂牌运营，四川军民融合高技术产业联盟和四川军民融合研究院顺利组建。全国唯一、规模20亿元的军民融合成果转化基金和规模5亿元的军民融合产业发展基金设立运行，3个军民融合金融服务中心成功组建。率先开展军民融合企业认定试点。

——这五年，我们坚持深化改革、扩大开放，经济社会发展增添新动力

重点领域改革取得阶段性成果。出台82个专项改革方案，应收账款融资服务等98项国省改革试点有序推进。“放管服”改革*实施，取消行政审批事项85项、行政审批前置条件556项、行政事业性收费123项。商事制度改革扎实推进，市场主体达到24.7万户。在全国率先开展县市区党政主要领导自然资源资产和生态环境责任离任审计试点。农村集体土地所有权、承包经营权、宅基地和集体建设用地使用权确权登记基本完成。不动产登记顺利实施。构建“1+15”国资国企改革政策体系，市属出资企业混合所有制比例达到70%以上。非公有制经济增加值占地区生产总值比重达到60.1%。新增金融机构47家，总数达到190家。新增上市挂牌企业23家，总数达到30家。

对外开放合作水平全面提升。连续四届高水平举办科博会，蝉联中国十佳品牌展会。引进爱默生等世界500强、华晨等国内500强企业30家，总数达到76家。到位国内省外资金1642亿元。引进国外智力项目57项，引进高层次人才、外籍专家416人次。实现外贸进出口总额124.2亿美元。到位外资总额、企业境外投资户数、对外投资总额三项指标均居全省第2位。与成渝地区、长三角、珠三角、京津冀和港澳台等区域合作不断深化，与欧美、日韩等地区交流日益频繁。国际友好城市达到22个。外事、台侨、口岸、海关、检验检疫等工作切实加强。

——这五年，我们坚持以人为本、共建共享，社会民生实现新进步

民生保障更加有力。安排民生资金570.8亿元，实施民生项目785个。城镇新增就业24.2万人，城镇登记失业率控制在4%以内。竣工保障性安居工程8.1万套，改造农村危旧房7568户。发放城乡低保、临时救助等资金34亿元，帮助特殊困难群众1959万人次。实施80周岁以上老人高龄津贴制度，惠及13万老人。下调社会保险缴费基数和费率，共计为参保者减轻负担6.1亿元。义务教育全面实行“三免一补”，农村义务教育学校学生免费营养午餐计划实现全覆盖。免除北川羌族自治县、平武县高中生学杂费。丘区农村脱贫攻坚示范区建设取得阶段性成效。全市脱贫55.3万人，贫困发生率下降到3.1%。生态环境持续改善，森林覆盖率从48.99%提高到53%，城区空气质量达标率达到83.2%，单位GDP能耗下降27.7%。对口支援阿坝州壤塘县、红原县工作扎实开展，援建雅安市雨城区灾后重建工作圆满完成。

社会事业全面发展。县市区全部通过义务教育基本均衡县国家级评估。全市高考本科硬上线人数和硬上线万人比连续16年全省“双第一”。高等院校达到14所，居全国地级市前列。成功承办全国第九届残运会暨第六届特奥会，三台县被命名为首批全国青少年校园足球试点县。医药卫生体制改革稳步推进，城乡医疗服务体系更加健全。完成国家重大传染病防治示范区项目建设，获评全国基层中医药工作先进单位。完善公共文化服务体系，城市15分钟、乡村30分钟公共文化服务圈基本形成。高水平承办第四届中国诗歌节，成功举办李白诗歌奖评选颁奖等大型文化活动。舞蹈诗剧《大北川》和电影《兰辉》等作品荣获全国大奖。广电、科普、史志、档案、统计、气象、水文、测绘等工作切实加强。工会、青少年、妇女儿童、残疾人、慈善、老龄等事业健康发展。

社会治理能力明显提升。“六五”普法圆满完成，荣获全国“六五”普法先进市。群众对社会治安满意度连续五年名列全省前2位。信访秩序更加规范。基层社区建设和乡村治理取得明显成效。违法建设整治扎实推进，拆除违法建筑86万平方米，整治面积171万平方米。安全生产工作切实加强，未发生重特大安全事故。食品安全状况满意率连续三年全省第一，被评为省级食品安全先进市。防震减灾和应急管理工作体系更加健全。民族宗教事务管理进一步加强。国防动员工作和后备力量建设持续加强，连续两届获得全国双拥模范城称号。国安、保密、邮政、住房公积金等工作积极推进。

——这五年，我们坚持依法行政、效能优先，政府建设取得新成效

权力运行更加规范。出台《绵阳市人民政府议事规则》《绵阳市重大行政决策程序规定》。市级部门行政权力目录编制完成，38个市级部门6274项“权力清单”向社会公布。成立市土地管理委员会、市公共资源交易管理委员会，土地出让、工程招投标等重点领域监管进一步加强。工作作风积极转变。*开展党的群众路线教育实践活动、“三严三实”专题教育和“两学一做”学习教育。扎实推进节庆论坛、楼堂馆所等专项整治行动，市本级累计压缩“三公”经费2675万元、下降19.2%。行政效能明显提升。20个市级部门热线并入市长热线，群众诉求得到及时回应。在全省率先推行建设项目全程并联审批。市本级办结行政审批事项135.2万件，办理提速70%。行政监督切实加强。建立市政府与市人大常委会、市政协工作联系机制，定期向市人大及其常委会报告、向市政协通报重大事项。1108件人大代表建议、2309件政协提案全部按时办结。深化廉政风险防控，强化行政监察和审计监督，政纪处分522人。

今年以来，我们牢牢把握稳中求进工作总基调，攻坚克难、真抓实干，经济发展平稳向好，社会大局和谐稳定。

经济运行平稳，发展势头良好。1—9月，实现地区生产总值1333.22亿元、增长8%，增速高于全省0.5个百分点，位居全省第6位，比去年同期提升2位。规上工业增加值增长9.9%，增速高于全省2个百分点，位居全省第6位，比去年同期提升1位。全社会固定资产投资918.7亿元、增长10.8%，增速位居全省第12位，比去年同期提升5位。社会消费品零售总额703.5亿元、增长12.4%，增速高于全省0.9个百分点。城乡居民人均可支配收入达到23740元、10176元，分别增长7.9%、9.7%。

结构持续优化，质量效益提升。1—9月，实现服务业增加值507.3亿元、增长8.7%，占地区生产总值比重同比提升1.5个百分点。八大重点产业产值增长9.7%，高于全市工业产值平均增速0.8个百分点。高新技术产业和战略性新兴产业产值占工业总产值比重分别达到53.3%、35.3%，同比分别提升1.8和1.4个百分点。工业企业产销率98.1%，高于全省1.4个百分点，同比提升0.9个百分点。完成地方一般公共预算收入81.23亿元，同口径增长11.8%，增速高于全省3.2个百分点。

改革纵深推进，招商成效显著。1—9月，供给侧结构性改革顺利推进，压减炼钢产能0.32万吨、炼铁产能8.4万吨。商品房销售面积增长15.9%，可供销售面积较2015年底减少133.5万平方米。规上工业企业产成品存货同比下降9.3%。规上工业企业资产负债率63.2%、同比下降5.9个百分点。全面创新改革试验开局良好，“放管服”改革*推进，国资国企、农业农村、投融资等领域改革取得阶段性成果。招商引资取得重大突破，新签约项目339个、投资金额1626.8亿元，分别增长28.4%、38.1%。国内省外履约项目479个、合同投资额2274.3亿元，分别增长72.3%、11.2%。国内省外到位资金337.1亿元、增长16.9%，增速居全省第1位，总量居全省第3位，实现全省排名连续5个季度上升。成功引进投资465亿元的京东方第六代柔性面板生产线等一批重大项目。

社会协调发展，民生持续改善。*推进脱贫攻坚工程，精准脱贫29130人，占年度目标80%。易地扶贫搬迁开工项目76个、建设住房426套，完工148套。扎实推进就业促进工程，城镇新增就业4.26万人，城镇失业人员再就业1.04万人。大力推进百姓安居工程，棚户区改造新开工8338套、占年度目标98.8%，基本建成10881套、占年度目标114.7%，棚改货币化安置4810套、占年度目标149.7%。持续推进教育助学工程，投入薄弱学校改造资金2738.2万元，实施项目覆盖学校62所，受益学生3.3万人。大力实施“雨露计划”，免费为336名贫困家庭子女进行职业技术培训。加快推进卫生惠民工程，全面启动城市公立医院综合改革，落实县级公立医院取消药品加成财政补助1490万元。减轻群众就医负担8520万元，县域内就诊率达到90%以上，新增82家社会办医疗机构。统筹推进社会保障工程，城乡居民基本养老保险参保人数达到219万人，9577名失地农民进入城镇职工基本养老保险。社保卡持卡人数新增23.1万人，达到274万人。安排资金6.2亿元，救助困难群众285.8万人次。

各位代表，五年来取得的巨大成就，是党中央国务院亲切关怀、省委省政府和市委坚强领导的结果，是市人大、市政协和人大代表、政协委员监督支持的结果，是全市广大干部群众奋力拼搏的结果。在此，我代表市人民政府，向全市各族人民，向人大代表、政协委员，向各民主党派、工商联、无党派人士和各人民团体，向所有关心支持绵阳改革发展的同志们及社会各界人士，向驻绵解放军指战员、武警官兵、政法干警和民兵预备役人员，向港澳台同胞、广大侨胞和海内外朋友们，表示诚挚的感谢并致以崇高的敬意!

在看到成绩的同时，我们也清醒认识到，全市经济社会发展还面临不少困难和问题，主要表现在：经济总量不大、质量不高，城乡发展不协调，县域发展不平衡；科技城建设市场化程度不高，军民深度融合的体制机制尚未完全建立；经济下行压力依然较大，财政收支矛盾突出；保障改善民生任重道远，基本公共服务均等化水平有待进一步提高；部分干部思想观念、能力水平、工作作风还不完全适应新形势新任务新要求，政府行政效能还需进一步提升。对这些问题，我们高度重视，正采取有力措施，切实加以解决。

未来五年工作建议大清相国

未来五年是全面建成小康社会的决胜阶段。绵阳既具备科技资源富集、产业基础坚实、开放优势明显、政策体系完备等加快发展的有利条件，也面临系统推进全面创新改革试验、成渝城市群发展、川陕革命老区振兴、长江经济带和一带一路建设等重大机遇。只要我们始终保持专注发展定力，坚持把发展作为第一要务、把经济建设作为兴市之要、把增进人民福祉作为不懈追求，坚持科学发展、加快发展工作取向，开拓创新、锐意进取，攻坚克难、真抓实干，就一定能推动绵阳发展再上新台阶、实现新跨越、铸就新辉煌！

今后五年政府工作的总体要求：高举中国特色社会主义伟大旗帜，以邓小平理论、“三个代表”重要思想、科学发展观为指导，*学习贯彻习近平总书记系列重要讲话精神，按照“四个全面”战略布局，践行“五大发展理念”，落实省委治蜀兴川方略，牢牢把握科学发展、加快发展工作取向，坚持创新驱动军民融合，坚持“两化”互动城乡统筹，坚持深化改革全面开放，在全省率先实现次级突破、率先全面建成小康社会，加快建设国家科技城和幸福美丽绵阳。

未来五年的主要预期目标：

——经济保持较快增长，质量效益稳步提高。地区生产总值年均增长8%以上，达到2500亿元。地方公共财政收入、城乡居民人均可支配收入增速高于地区生产总值增速。工业效益进一步提升。县域经济加快发展，城镇化率达到57.9%。现代产业体系和现代基础设施体系基本形成。

——创新能力不断增强，发展方式明显转变。高质量完成全创试验，建成国家军民融合创新示范基地。科技进步综合水平指数达到70%，高新技术产业和战略性新兴产业产值占工业总产值比重分别达到60%、40%以上。科技城建设实现创新型、开放式、突破性发展。

——生态环境总体改善，人与自然更加和谐。森林覆盖率、城市绿地率分别达到54%和38%以上。生产生活方式绿色、低碳水平显著提升，单位GDP能耗、主要污染物、二氧化碳排放、流域出境断面水质达到国控、省控目标。

——改革开放*推进，市场活力持续迸发。重点领域和关键环节改革取得决定性成果，形成一批可复制、可推广的绵阳经验。市场化改革取得较大进展，重点改革任务全面完成，开放型经济新体制基本形成，建成西部内陆改革开放新高地。

——社会事业全面进步，幸福指数持续提升。提前一年完成脱贫攻坚任务。教育、体育、卫生、文化事业全面发展，建成更加公平可持续的社会保障体系，基本实现城乡公共服务均等化，幸福绵阳建设取得新成效。

为实现上述目标，要重点抓好以下几方面工作。

一、坚持创新发展，提高发展质量效益

坚持把创新发展作为面向未来的核心战略，作为推动经济转型的主要动力，推动经济更高质量、更有效率、更加公平、更可持续发展。

系统推进全面创新改革试验。以实现创新驱动发展为目标，以推动科技创新为核心，以军民融合发展为特色，以破除体制机制障碍为主攻方向，依托绵阳科技城建设，开展系统性、整体性、协同性改革的先行先试，积极构建军民融合“五大体系”，打造国家军民融合创新示范基地。支持空气动力新城、航空新城、科学新城建设世界一流科技创新基地。研究与试验发展经费支出占地区生产总值比重保持全国领先水平。加快军民两用技术协同创新平台建设，用好国家军民两用技术交易中心，着力打通“军转民”“民参军”双向互动通道。推进科技成果使用权、处置权、收益权改革和股权激励试点。办好四川军民融合研究院、中国国际人才市场国家科技城（绵阳）分市场，推动西南科技大学等在绵高校培养军民融合研究型、应用型、技能型人才。加强与央属军工集团合作，争取在绵布局重大项目，建好磁性材料产业园、核技术应用产业园等军民融合产业园区，着力打造一批军民融合高技术产业集群。力争科技型中小企业超过1万家。

着力扩大投资消费。充分发挥投资对稳增长的关键作用，坚持以项目为中心组织经济工作，确保全社会固定资产投资年均增长8%以上。突出抓好工业投资和技改投资，力争投资增速高于全社会固定资产投资增速。认真落实鼓励民间投资政策，大力推广PPP模式，引导社会资本进入基础设施和公共服务领域。认真落实《绵阳市2016—2020项目计划》，持续策划、包装、储备项目，充实完善项目库并实行动态管理。狠抓重大产业项目、重大基础设施项目等省市重点项目建设，形成经济社会发展有力支撑。充分发挥消费对稳增长的基础作用，积极推进国家信息消费试点市建设，加快培育服务消费、时尚消费和农村消费。办好房交会、国际汽车展等展会，积极参与全省市场拓展“三大活动”。力争社会消费品零售总额年均增长11%。

加快构建现代产业体系。围绕产业转型升级、振兴实体经济，加快实施“两个一号工程”，积极构建创新能力强、品质服务优、协作紧密、环境友好的现代产业新体系。优先发展工业。坚持把工业作为三次产业协调发展的核心和枢纽，积极对接“中国制造2025四川行动计划”，*实施“工业强市”战略，加快建设西部先进制造强市。认真落实《工业重点产业及空间布局方案》，大力发展高新技术产业和战略性新兴产业，着力培育2000亿级电子信息产业，300亿级汽车、新材料、节能环保产业，200亿级高端装备制造及生物产业。加快食品、化工等传统工业升级改造，推动其向产业链、价值链高端延展。实施工业大企业大集团培育“领航计划”，支持中小微企业“专精特新”发展，构建“大企业顶天立地、小企业铺天盖地”的发展格局，形成业态高端、梯次衔接的现代企业集群，力争规模以上工业企业突破1300户，上市挂牌企业达到100户。大力实施“51025”重点园区培育计划，推动电子信息产业园、汽车产业园等工业园区专业化、特色化、高端化发展，培育2000亿级园区1个、1000亿级园区1个、100—500亿级园区6个，园区产业集中度达到85%。同步发展现代服务业。认真落实《现代服务业重点产业及空间布局方案》，培育打造50个重点项目、100户重点企业、30个重点品牌，推动更多项目、企业和品牌进入全省服务业“三百工程”行列，推动电子商务、物流、旅游、金融、科技服务、会展、文化创意、健康养老等八大重点产业加快发展。力争电子商务交易额年均增长30%，旅游总收入年均增长18%，文化产业增加值、物流业增加值、会展业收入年均增长10%以上。力争养老服务设施覆盖90%以上城市社区、75%以上乡镇和50%以上农村社区。促进批发、零售、住宿、餐饮等传统服务业转型升级，提高便利化、精细化、品质化水平。力争服务业增加值占地区生产总值比重达到43%，加快建成现代服务业强市。大力发展现代农业。加快构建现代农业产业体系、生产体系、经营体系、物质装备体系，提高农业质量效益和竞争力。大力推进“沃野绵州”现代生态循环农业工程建设，积极发展都市农业、休闲农业，培育一批无公害农产品、绿色食品、有机食品，建成2个国家级生态原产地产品保护示范区，争取三台“涪城麦冬”列入国家中药材大品种。加大全国、全省现代农业林业畜牧业重点县建设力度，推进国家级杂交水稻制种基地和绵阳国家农业科技园建设，建成现代农业（林业）示范园10个，高标准农田133万亩。加大新型农业经营主体培育力度，新增农民专业合作社1000家、家庭农场（林场）1500个、市级以上农业产业化重点龙头企业120家。落实强农惠农富农政策，持续增加农民收入。

深化重点领域改革。坚持分类指导去产能、长短结合去库存、积极稳妥去杠杆、多措并举降成本、对标小康补短板。进一步压减钢铁、化工、水泥等行业过剩产能，棚户区改造货币化安置比例达到50%以上，进一步降低企业制度**易成本、税费成本、融资成本、要素成本和经营成本。以提高国有资本效率、增强国有企业活力为中心，分类推进国有企业改革，不断增强国有经济活力、控制力、影响力和抗风险能力；聚焦农村集体产权制度、农业经营制度、农业支持保护制度、城乡发展一体化体制机制和农村社会治理制度五大领域，深化农业农村综合改革；推进户籍制度改革，积极实施居住证制度；以优化金融服务、促进实体经济发展为重点，推进投融资体制改革。建立完善容错纠错机制，营造支持改革、鼓励创新、宽容失败的政策环境和舆论氛围，不断把改革红利转化为发展新动能和民生新福祉。

二、坚持协调发展，形成平衡发展格局

坚持区域协同、城乡统筹，在协调发展中拓展发展空间、增强发展后劲。

大力推进新型城镇化。抓好国家新型城镇化综合试点，健全城乡发展一体化体制机制，推动城镇公共服务向农村延伸，逐步实现城乡基本公共服务制度并轨、标准统一。完成城市总体规划修编工作，推动安州区加快融入主城区，努力拓展城市空间、优化城市布局、完善城市功能、提升城市品质，增强城市发展的全局性、系统性、持续性、宜居性。力争中心城区建成区面积达到175平方公里，常住人口达到175万人，努力建设和谐宜居、富有活力、具有特色的现代化区域性中心城市。支持江油市、三台县建设50平方公里、50万人口中等城市，支持梓潼县、盐亭县、平武县、北川羌族自治县加快建设特色精品县城。大力推动特色小城镇建设，打造一批产业特而强、功能聚而合、形态精而美、体制活而新的工业重镇、商贸强镇、文化古镇和旅游名镇。开展农村人居环境整治行动。加大历史文化名村名镇和传统村落保护力度。按照“小规模、组团式、微田园、生态化”要求，建成2600个以上幸福美丽新村。

发展振兴县域经济。县域经济既是全市经济发展的短板，也是经济增长的潜力所在。把振兴县域经济摆在更加突出的位置，强化全域统筹，注重分类指导，增强县域发展能力，统筹推进平原、丘陵、山区同步发展，推动形成要素有序自由流动、主体功能约束有效、基本公共服务均等、资源环境可承载的区域经济协调发展格局。支持涪城区、游仙区、安州区、江油市等重点开发区，发展先进制造业和现代服务业。支持三台县、梓潼县、盐亭县等农产品主产区，推进农业现代化，因地制宜发展特色工业和服务业。支持平武县、北川羌族自治县等重点生态功能区，加强资源保护利用，积极发展现代旅游业。力争县市区地区生产总值达到1900亿元、占全市经济总量80%以上，各县市区经济综合实力在全省类区县排名实现较大提升。

加快构建现代基础设施体系。突出抓好建设领域三大攻坚行动，协同推进水利、能源、信息等基础设施建设，形成安全高效、智能绿色、互联互通的现代基础设施网络。大力开展城建攻坚行动，积极推进城市轨道交通项目，完成“重构一环、贯通二环”等市政道路工程，沉抗水厂、平政雨水提升站等给排水工程，三江六岸改造提升等城市景观工程。加快海绵城市和地下综合管廊建设，新建一批城市公园和停车场。抓好城乡环境特别是背街小巷和城郊接合部综合整治。打好交通攻坚大会战，加快构建西部区域性综合交通枢纽。建成成兰铁路、西成客专、绵西高速，加快建设九绵高速、绵苍高速、广平高速，规划建设绵遂铁路、绵中高速、三大高速，形成“一纵一横两联”铁路主骨架和“一环八射三联”高速公路网。改造国省干线公路，提升农村公路建管水平。加快推进绵阳机场改扩建工程和通航机场建设。扎实开展科技城集中发展区建设攻坚行动，抓好骨干道路等重大市政基础设施和公共服务配套基础设施建设，确保30平方公里核心区基本建成，100平方公里主体框架基本形成，打造全市经济发展新引擎和宜居宜业新城区，有效拓展绵阳城市和产业发展空间。基本建成武引二期灌区、开茂水库、沉水水库、金峰水库等大中型水利工程，开工建设草溪河水库、祠堂湾水库、建兴水库等中型水利工程。加快骨干电网、燃气管网等能源基础设施建设。*推进国家智慧城市试点市和宽带中国示范城市建设，构建高速、移动、安全、泛在的新一代信息基础设施。

三、坚持绿色发展，积极改善生态环境

把绿色发展融入经济社会发展各方面、全过程，让绵阳天更蓝、地更绿、水更清、环境更优美。促进资源集约利用。严守资源消耗上限、环境质量底线、生态保护红线，实施最严格的耕地保护制度，大幅提高资源利用综合效益。推进绿色生产，支持节能环保、清洁能源等产业发展，加强风电、生物质发电等新能源研发应用，加快建设科技城循环经济产业园等示范基地，进一步提升非化石能源占一次能源消费比重。实施循环发展引领行动计划，推进工业“三废”回收综合利用。全面实施燃煤锅炉节能环保综合提升工程。力争万元工业增加值用水量下降23%，单位GDP碳排放下降15%。积极开展效能“领跑者”行动、节约型公共机构示范创建活动，推动全民在衣食住行游等方面向绿色低碳、文明健康方式转变。

加大环境治理力度。建立健全政府、企业、公众共治的环境治理体系，打好大气、水、土壤污染防治“三大战役”，推进污染物综合防治和环境治理。完善大气污染联防联控机制，坚持“减排、抑尘、压煤、治车、控秸”，力争PM2.5年均浓度值比2015年下降18%，城区空气质量达标率达到85.5%。坚持生产、生活、生态用水“三水共治”，持续推进小流域综合治理，县以上城市饮用水源水质全面达标，乡镇污水处理设施实现全覆盖。强化土壤重金属污染防控，保持土壤环境质量总体稳定。

切实加强生态建设。以生态建设为抓手，加快建设美丽绵阳。积极推进生态文明先行示范区、国家森林城市建设，努力创建国家生态园林城市。开展大规模绿化绵州行动，实施天然林资源保护和森林质量提升工程，力争营造林30万亩。系统推进涪江、安昌江流域生态系统建设和水环境保护，加强河湖库区湿地保护，建好三江湖等湿地公园。加强生物多样性保护，推进王朗、雪宝顶、小寨子沟、千佛山等国家级自然保护区建设。完善县市区、园区党政主要领导自然资源资产和生态环境责任离任审计制度，继续实施涪江流域水环境生态补偿和水质超标资金扣缴制度，探索更多可复制、可推广的绿色发展绵阳经验。

四、坚持开放发展，着力实现合作共赢

坚持内外需协调、引进来和走出去并重，全方位扩大对外开放，打造内陆开放型经济新高地。

全力抓好招商引资。把“狠抓招商引资扩增量”作为扩大对外开放的首要任务。积极申建综合保税区，加快中德产业创新合作平台建设，打造绵阳外向型经济发展主平台。办好科博会、中国电子商务发展峰会，积极参加西博会、中国知名企业四川行等活动。完善提升县市区、园区“一把手”外出招商和市县联动招商机制，进一步完善招商激励政策，持续开展精准专业招商。坚持“招新引优、招大引强、招才引智”，突出抓好重大产业化项目招商，强化项目履约。力争累计引进5亿元以上项目200个，到位内资2400亿元，利用外资10亿美元以上。

大力实施走出去战略。对接国家内贸流通“五年发展规划”，加大“绵阳造”产品推介力度，支持重点企业拓展市场。创新外贸发展模式，实施“自主品牌出口增长”计划，力争外贸进出口总额年均增长8%。鼓励长虹、九洲等优势企业参与“一带一路”沿线国家及地区产能合作和重大设施建设，推动装备、技术、标准、服务走出去，深度融入全球创新链、产业链、价值链。

全面深化区域合作。主动融入“一带一路”和长江经济带建设，加强与长三角、珠三角、京津冀和港澳台等区域合作，深度参与成渝经济区、成都平原经济区和川陕革命老区协作发展，打造成德绵协同创新共同体。稳步提升与欧美、日韩等地区合作水平，扩大与非洲、中东、东南亚等地的新兴市场国家交流，发展更多国际友好城市。

五、坚持共享发展，努力增进人民福祉

按照“人人参与、人人尽力、人人享有”的要求和“坚守底线、突出重点、完善制度、引导预期”的原则，保障改善民生，提高人民生活水平，加快建设幸福绵阳。

坚决打赢脱贫攻坚战。推动农村贫困人口全面脱贫是补齐全面建成小康社会短板的重中之重。坚持以三大片区为主战场，大力推进丘区农村脱贫攻坚示范区建设，打好精准脱贫“3+10+N”组合拳，落实“六个精准”“四个一批”要求，着力解决住房建设、增加收入、教育保障、医疗保障等问题，实现农村贫困人口“两不愁三保障”和“四个好”目标。整合扶贫资源，构建专项扶贫、行业扶贫、社会扶贫“三位一体”大扶贫工作格局。确保现行标准下贫困人口全部脱贫、贫困村和贫困县全部“摘帽”。

*实施民生工程。确保民生投入占公共财政支出比重达到63%以上。*实施就业优先战略，抓好高校毕业生、农村转移劳动力、就业困难人员和失业人员等重点人群就业和再就业，城镇新增就业21.6万人，城镇登记失业率控制在4%以内。改造棚户区危旧房2.2万套。实施全民参保计划，城乡居民基本养老保险参保覆盖面达到87%。稳定市场物价，确保有效供给。推进社会救助，提高城乡低保水平，加强对特殊困难群众的精准帮扶，关心关爱农村留守儿童。发展慈善事业，深化“量体裁衣”式残疾人服务。扎实做好援藏、援彝工作。

大力发展社会事业。坚持立德树人，全面实施素质教育，普及学前三年教育，巩固义务教育均衡发展成果，提升高中教育质量，积极发展职业教育，鼓励社会力量办学，努力建设全国教育强市。落实全民健身战略，大力开展“三大球”活动，推动学校体育、竞技体育、社会体育协调发展，培育壮大体育产业，加快建成西部体育强市。践行社会主义核心价值观，全面提升市民文明素质。繁荣文化事业，发展文化产业，建设西部文化强市。落实“健康中国2030”规划纲要，深化公立医院综合改革，完善分级诊疗等五项基本医疗卫生制度，做好人口和计划生育工作，大力发展中医药事业，支持社会力量办医，全方位、全周期保障人民健康，努力建设健康绵阳。*实施哲学社会科学创新工程。积极发展妇女儿童、老龄事业，继续做好外事侨务、新闻出版、广播影视、统计、档案、地方志等工作。

提高社会治理水平。健全城市管理体制，推广网格化管理服务，增强社区服务功能，实现政府治理和社会调节、居民自治良性互动，着力提升城市管理水平。切实治理城区交通拥堵。大力开展“七五”普法，深化“法律七进”，提升公共法律服务水平，建设法治绵阳。落实重大决策社会稳定风险评估机制，打造“阳光信访、责任信访、法治信访”，健全治安防控“七张网”，依法打击各类违法犯罪活动，推进“打非治违”和安全生产专项整治，坚决遏制重特大事故发生，建设平安绵阳。积极推进诚信体系建设，建成覆盖全社会的征信系统。依法处置非法集资，确保不发生区域性金融风险。实施最严格的食品药品安全监管，加快创建国家食品安全城市。抓好气象、防震减灾、水文监测预报和地质灾害防治，有效防范和应对重大自然灾害。加强应急管理，提升突发事件处置能力。贯彻党的民族政策,推进宗教事务依法管理。加强国防动员和后备力量建设，支持国防和军队改革，做好双拥共建、人民防空等工作。

建设人民满意政府永远在路上。未来五年，我们将认真落实《法治政府建设实施纲要（2015—2020年）》，健全完善重大行政决策机制，自觉接受市人大及其常委会法律监督和市政协民主监督，加强政府诚信建设，提高政府公信力，努力建设法治政府。我们将*推进“放管服”改革，大力实施“互联网+政务服务”，在全省率先将相对集中行政许可权试点扩大到所有县市区，让企业和群众少跑腿、好办事、不添堵，努力建设服务型政府。我们将严格执行中央、省、市转变作风各项规定，厉行节约、反对浪费，进一步精简会议和文件，加强财政资金管理，严控“三公”经费支出。强化正风肃纪和审计监督，从严惩治腐败，努力建设廉洁政府。

各位代表！绵阳发展已经站在新的历史起点上。回顾过去，我们栉风沐雨；展望未来，我们豪情满怀。让我们紧密团结在以习近平同志为核心的党中央周围，在省委省政府和市委坚强领导下，开拓创新、奋发有为，攻坚克难、拼搏实干，为加快建设国家科技城和幸福美丽绵阳而努力奋斗！
